# Influence of breast milk microbiota on the composition of the early intestinal microbial community in goat kids: a study of composition and correlations

**DOI:** 10.1128/spectrum.02642-24

**Published:** 2025-10-20

**Authors:** Lanmuyi Gou, Gan Luo, Zihan Xia, Wentao Zhang, Shenglin Li, Kegu Ji'e, Taichun Gao, Kehamo Abi, Falong Yang

**Affiliations:** 1 College of Animal & Veterinary Sciences, Southwest Minzu University 66336, Chengdu, China; USDA-ARS Arkansas Children's Nutrition Center, Little Rock, Arkansas, USA

**Keywords:** goat, breast milk, goat kid, gut microbiota, probiotics

## Abstract

Colonization of gut microbes in young ruminants plays a fundamental role in their lifelong health. Although the relationship between breast milk (BM) and the gut microbiota of young animals has been reported, its specific impact on goats has yet to be thoroughly explored. Therefore, this study aimed to comprehensively reveal the influence of breast milk feeding on the development of intestinal microorganisms in goat kids and the interactions between breast milk microbiome and fecal microbiome. The results showed that there was a significant difference in the richness of breast milk microbiota between the BMD1, BMD7, and BMD14 groups (*P* < 0.05). At the genus level, the bacterial microbiota in breast milk across different days of age was predominantly composed of *Pseudomonas*, *Asticcacaulis*, *Mannheimia*, *Sphingomonas*, and *Staphylococcus*. In contrast, the fecal microbiota of goat kids was primarily dominated by *Escherichia-Shigella*, *Butyricicoccus*, *Bacteroides*, *Lactobacillus*, and *Limosilactobacillus*. Significant correlations (*P* < 0.05) were observed between the microbial communities of breast milk and the fecal microbiota of goat kids. Specifically, *Delftia* in breast milk was positively correlated with *Limosilactobacillus* and *Lactobacillus* and negatively correlated with *Lachnoclostridium* in the rectal feces of goat kids, and *Lactobacillus* in breast milk was positively correlated with *Ruminococcus_gnavus_group*. These results suggest that the structure of the breast milk microbiota may influence the colonization of the intestinal microbiota in goat kids, especially for some probiotic genera such as *Lactobacillus*. In conclusion, this study elucidates the dynamic changes in breast milk microbiota and the shifts in goat kid fecal microbiota following breast milk feeding, as well as the correlations between them. These findings provide a scientific foundation for enhancing goat kid growth, development, and health through early microbiota modulation in practical production.

**IMPORTANCE:**

The gastrointestinal (GI) microbiota has a profound effect on host health, especially in resisting pathogen colonization and promoting intestinal function (T. Zhong, Y. Wang, X. Wang, A. Freitas-de-Melo, et al., Front Microbiol 13:1020657, 2022, https://doi.org/10.3389/fmicb.2022.1020657). The results show that the stability of rumen microbial communities and their associated functions in lambs is not achieved until they reach at least 20 days of age (B. Brooks, B. A. Firek, C. S. Miller, I. Sharon, et al., Microbiome 2, 2014, https://doi.org/10.1186/2049-2618-2-1). From a nutritional perspective, young animals, such as lambs, can be considered non-ruminants (Y. Li, L. Ren, Y. Wang, J. Li, et al., Nutrients, 14, 2022, https://doi.org/10.1017/S1751731119003148). The gut is not only a key organ for digestion and absorption of nutrients, but also plays a variety of important roles in maintaining overall health. Thus, udder feeding may have a significant effect on the construction of the gut microbiota in lambs until 20 days of age. Although the relationship between breast milk (BM) and the gut microbiota of young animals has been reported, its specific impact on goats has yet to be thoroughly explored. Therefore, this study aimed to comprehensively reveal the influence of breast milk feeding on the development of intestinal microorganisms in goat kids and the interaction relationships between breast milk microbiome and fecal microbiome.

## INTRODUCTION

As a livestock animal of great economic value worldwide, goats play an indispensable role in human life by providing products such as meat, dairy products, and fur (1–3). According to the Food and Agriculture Organization of the United Nations, there are about 1,000 goat breeds and more than 830 million goats in the world, of which China has one of the largest populations of goats in the world, with about 140 million goats (4). With the development of society, the demand for milk and meat products is expanding. Although intensive farming can meet human demand for livestock products, the low degree of standardized feeding management in actual production leads to increased morbidity and mortality in goats (5). In particular, the stress syndrome caused by early weaning of lambs in the growth process leads to diarrhea, which in severe cases leads to the death of the lambs, causing significant economic losses to the large-scale breeding industry (6). Ensuring the health of goat kids is therefore essential to the sustainability of goat farming.

A growing body of research suggests that the gut microbiota is a large and complex organic assemblage that is closely linked to the health of the host. The gastrointestinal (GI) microbiota has a profound effect on host health, especially in resisting pathogen colonization and promoting intestinal function (7, 8). The colonization of the gastrointestinal tract of newborn lambs begins at birth, and the succession process continues until the microbiota reaches a stable state later in life (9). At present, the preponderance of research concerning the gastrointestinal microbiota in goat kids has been directed toward the rumen microbiota, while studies on the intestinal microbiota of goat kids remain relatively scarce. Nevertheless, recent scholarly findings indicate that the stability of rumen microbial communities and their associated functions in lambs is not achieved until they reach at least 20 days of age (10). Consequently, from a nutritional perspective, young animals, such as goat kids, can be considered non-ruminants. The gut microbiota has a significant impact on the physiological and immunological development of lambs during the critical pre-weaning period, especially before the age of 20 days (10). At the same time, maternal microbes influence the development of the intestinal immune system already during fetal life. Various factors influence the development of the lamb’s gut microbiota, including birth method, antibiotic use, environmental exposure, and diet—particularly breastfeeding (11, 12). Therefore, further studies are needed to investigate the relationship between breast milk and goat kid gut microorganisms, especially the species of vertically transmitted microorganisms.

Breast milk is considered to be the most optimal nutrient for the normal growth of goat kids and is the key to acquiring immunity and enhancing physical fitness, as well as improving the survival rate. In particular, colostrum contains significant amounts of immunoglobulins, lactoferrin, and various types of growth factors, which provide essential protection to newborn lambs through passive immunity (13). For neonatal ruminants, colostrum intake is the sole source of immunoglobulins (Ig) during the first month after birth and is crucial for the establishment of the early gut microbiota in lambs (14). Studies have shown that the breast milk microbiota has a significant impact on the development of the infant gut microbiota, particularly through key probiotics such as *Lactobacillus*, *Bifidobacterium*, and *Enterobacter* (15). Another study indicated early feeding of colostrum to calves after birth facilitates the formation of gut microbial diversity, and there is a positive correlation between *Bacteriaceae* in the foal gut microbiota and serine/glycine in breast milk (16, 17). Ruminants possess a specialized rumen digestive system, which plays a fundamental role in their growth and development. Furthermore, the complex microbial community within the gut of ruminants is essential for digestion, metabolism, nutrient compensation, and overall physiological development of the host (18). However, current research on the composition and structure of gut microbiota in early-stage goat kids and its correlation with breast milk is not yet clear.

The present study focuses on goat breast milk and their goat kids, using 16S rRNA gene sequencing technology to investigate the dynamic changes in the composition and structure of the milk and goat kid gut microbiota and conducts correlation analysis between the composition and structure of the milk microbiota at different periods and the goat kid gut microbiota. The aim is to clarify the impact of breastfeeding on the early gut microbiota of goat kids. Thus, promoting growth and development of goat kids by interfering with the structure of the intestinal microbiota in practice production.

## MATERIALS AND METHODS

### Experimental design and sample collection

The experiment was conducted at Chuanzong Goat Industry Co. Ltd., located in Lezhi County, Sichuan Province, China. A completely randomized design was used to select five healthy Chuanzhong black goats (Lezhi type) that met the following criteria: no history of antibiotic treatment, similar parity, and an average body weight of 42 ± 0.7 kg (Table 1). Breast milk (BM) samples (~50 mL each) were aseptically collected at five postpartum time points: 1 day (BMD1), 3 days (BMD3), 7 days (BMD7), 14 days (BMD14), and 21 days (BMD21). For milk collection, the udder was first wiped with an alcohol swab, the teats were then immersed in sterile iodine solution, and finally rinsed with sterile saline. The first few streams of milk were discarded to minimize contamination. Subsequently, approximately 50 mL of milk was collected into sterile centrifuge tubes and subjected to somatic cell count (SCC) testing using a BacSomatic analyzer (FOSS, Hillerød, Denmark). At the same time, the samples were flash-frozen in liquid nitrogen and stored at −80°C until DNA extraction for microbial composition analysis. The SCC values of all samples were below 3.1 × 10⁵ cells/mL, confirming the absence of subclinical mastitis (19).

**TABLE 1:** Body weight data of goats

Earphone number	Body weight (kg)
1322	43.7
1260	42.4
1944	41.9
1044	44.3
1249	41.2

**Fig 1:**
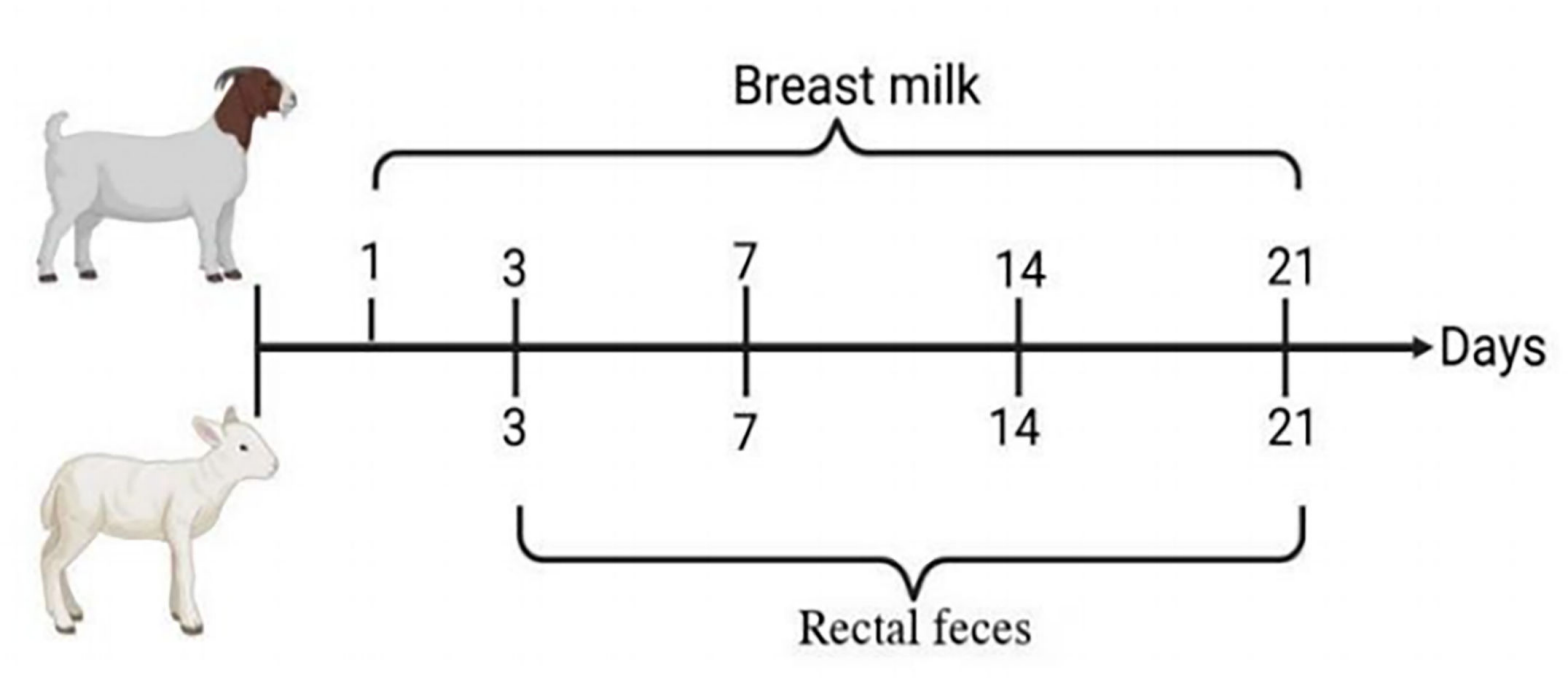
Sampling flow chart.

Meanwhile, rectal feces samples were collected from five healthy goat kids at four time points: 3 days (FD3), 7 days (FD7), 14 days (FD14), and 21 days (FD21). For fecal collection, fresh feces (approximately 20 g) were collected directly from the rectum using disposable sterile polyethylene (PE) gloves, by gently inserting the middle and index fingers near the rectal opening. The samples were immediately transferred into sterile cryovials, flash-frozen in liquid nitrogen, and subsequently stored at −80°C until further analysis. A schematic overview of the sampling process is provided in attached Fig. 1.

The composition of the feed is shown in Table 2.

**TABLE 2:** Feed ingredients of goats

Item	Experimental diet (%)
Ingredients	
Corn	60
Soybean meal	10
Cotton meal	12
Fish meal	1
Bran	11
NaCl	1
Premix	5

### DNA extraction, PCR amplification, and high-throughput sequencing

Breast milk samples and rectal fecal samples of goat kids were transported on dry ice to Shanghai Personal Biotechnology Co., Ltd. (Shanghai, China) for DNA extraction and 16S sequencing. The breast milk samples' DNA and rectal fecal samples' DNA were extracted using the Mag-Bind Soil DNA Kit (M5635-02) (Omega Bio-Tek, Norcross, GA). And the 16S rRNA sequence was used for amplification, sequencing, and analysis. The primers were designed according to the conserved regions in the sequences, and the sample-specific barcode sequences were added for PCR amplification of the rRNA variable regions V3–V4. The primers specific to the V3–V4 region of bacterial 16S rRNA were used for PCR amplification, 338F (5′-ACTCCTACGGGGAGGCAGCA-3′), 806R (5′-ACTCCTACGGGAGGCAGCA-3′). PCR amplicons were purified with Vazyme VAHTSTM DNA Clean Beads (Vazyme, Nanjing, China) and quantified using the Quant-iT PicoGreen dsDNA Assay Kit (Invitrogen, Carlsbad, CA, USA). Based on the fluorescence quantification results, the samples were mixed according to the sequencing volume required for each sample. The TruSeq Nano DNA LT Library Prep Kit (Illumina, Inc., San Diego, USA) from Illumina was used for library construction. Finally, the highly variable V3–V4 region of the bacterial 16S rRNA gene, which is about 430 bp in length, was selected for sequencing. Sequencing data generated in this study have been deposited in the NCBI Sequence Read Archive database under BioProject accession number PRJNA1255846: http://www.ncbi.nlm.nih.gov/bioproject/1255846.

### Bioinformatics analysis of sequence data

In the present study, bioinformatics analysis was conducted utilizing QIIME2 version 2019.4. The raw sequencing data were initially processed with the demux plugin. Subsequently, primer excision was executed using the cutadapt plugin, followed by a suite of data processing steps including quality filtering, denoising, sequence splicing, and chimera detection and removal, all facilitated by the DADA2 plugin. Non-singleton amplicon sequence variants (ASVs) were aligned with mafft. QIIME2 was further employed to calculate a panel of diversity indices for each sample, encompassing the Chao1, Observed Species, Shannon, and Simpson indices. The box plot was utilized to graphically represent the abundance and evenness of ASV among samples. Beta diversity analyses were performed using the UniFrac distance metric to assess the variation in microbial community structure. These analyses were implemented with R and QIIME2 software, and the results were visualized via non-metric multidimensional scaling (NMDS). QIIME2 was also applied to generate taxonomic composition and abundance tables at six hierarchical levels: phylum, class, order, family, genus, and species. The outcomes were depicted in bar charts for each sample. Lastly, the linear discriminant analysis effect size (LEfSe) method was implemented to discern differentially abundant taxonomic units between the groups.

### Statistical analysis

Body weight data were expressed as mean ± SE. The Shannon and Simpson diversity indices were used to assess species richness and distribution evenness in the microbial community. Spearman’s rank correlation coefficient was applied to examine the relationship between microbial composition in breast milk and rectal feces of goat kids. Differences in the abundance of coexisting probiotics in breast milk and rectal fecal samples were analyzed using the Kruskal-Wallis non-parametric test. A *P* value of <0.05 was considered statistically significant.

## RESULTS

### Diversity analysis of breast milk and goat kid fecal microorganisms

**Fig 2:**
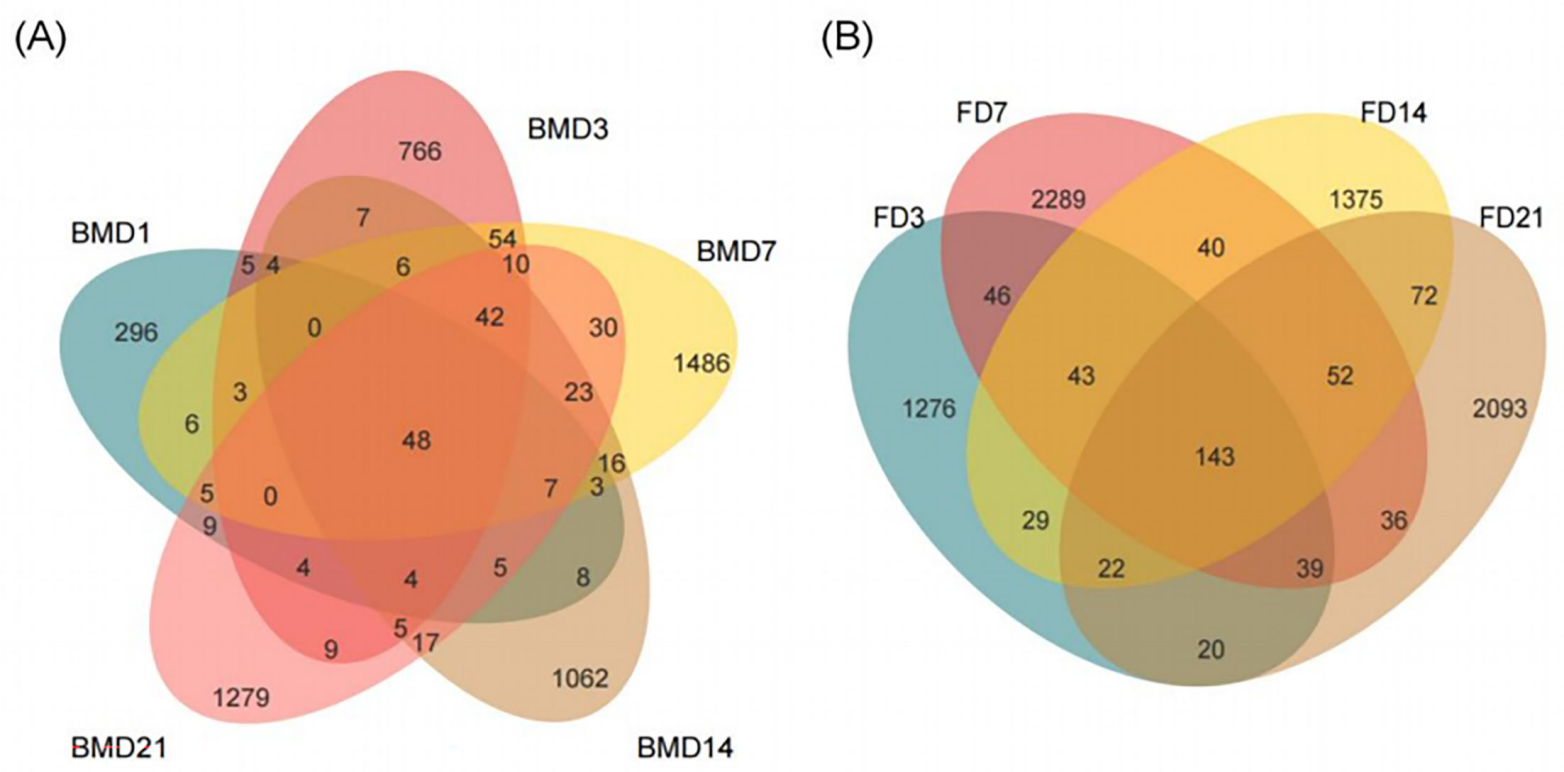
(**A**) Venn diagram of the number of ASVs of bacteria in the breast milk. (**B**) The Venn diagram represents the distribution of ASVs of bacteria identified in the rectal feces of goat kids. Five per group per time point (n = 5).

To ascertain the species shared and unique across various time points, a community analysis was conducted employing a Venn diagram, based on the abundance values of ASVs. Breast milk samples from different time points (BMD1, BMD3, BMD7, BMD14, BMD21) contained 296, 766, 1,062, 1,486, and 1,279 unique ASVs, respectively, with 48 ASVs shared among all five groups (Fig. 2A). In goat kid fecal samples (FD3, FD7, FD14, FD21), we identified 1,276, 2,289, 1,375, and 2,093 unique ASVs, with 143 ASVs shared across all four groups (Fig. 2B). This study demonstrates differences in microbiota composition in both breast milk and in the feces of goat kids at different ages.

**Fig 3:**
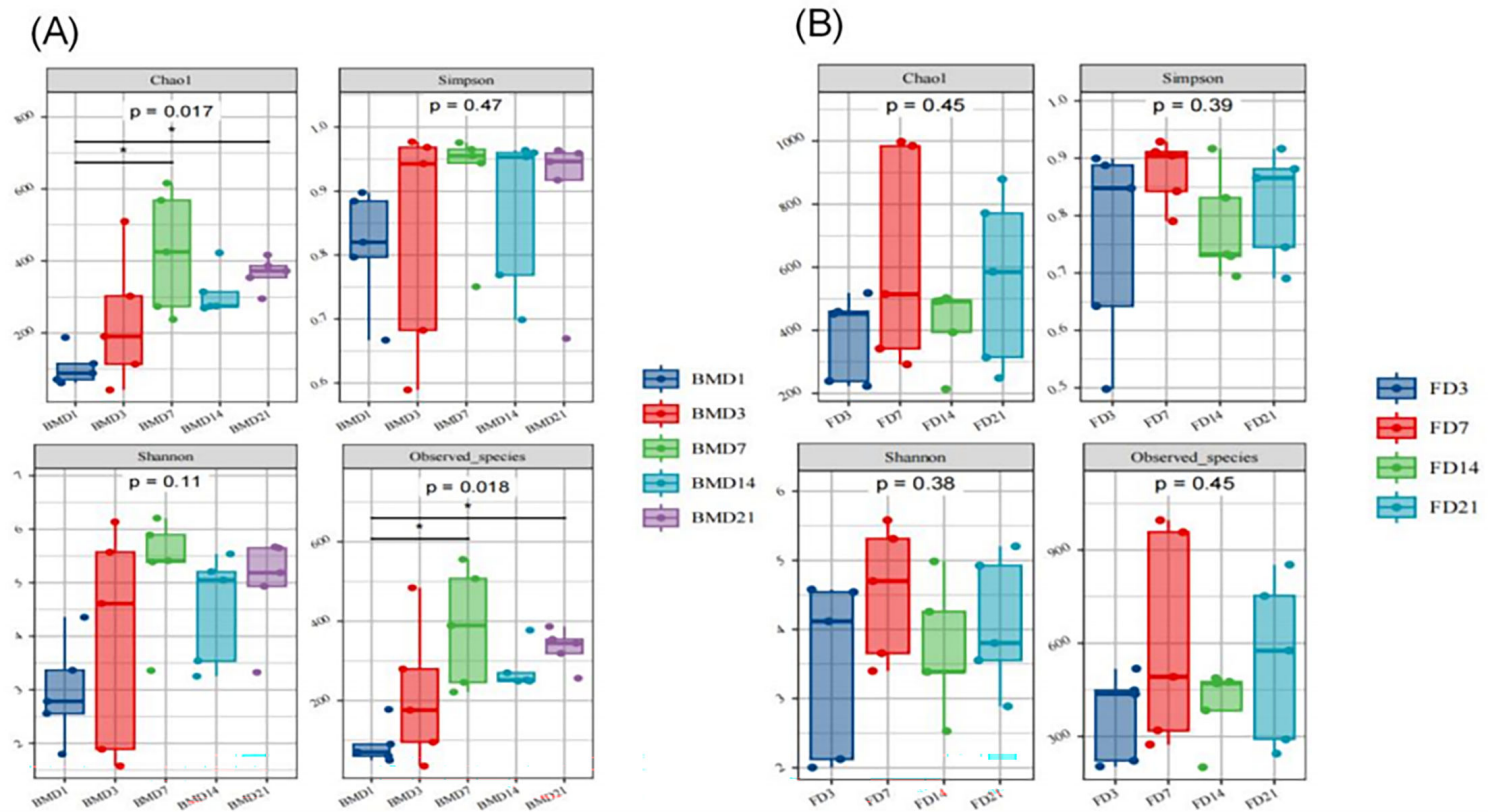
(**A**) Alpha diversity of breast milk microbiota. (**B**) Rectal fecal alpha diversity in goat kids. Abundance index: Chao1 and Observed species indices. Diversity index: Shannon and Simpson indices, **P < 0.01, **P < 0.001. Five per group per time point (n = 5).

Alpha diversity analysis of the breast milk samples revealed significant differences in microbial richness between the BMD1 and BMD7 groups (*P* < 0.05), as well as between the BMD1 and BMD21 groups (*P* < 0.05). However, there was no significant difference in diversity indices (Shannon and Simpson) between groups (*P* > 0.05) (Fig. 3A). In goat kid fecal samples, no significant difference in alpha diversity indices was observed across the different time points (*P* > 0.05) (Fig. 3B).

**Fig 4:**
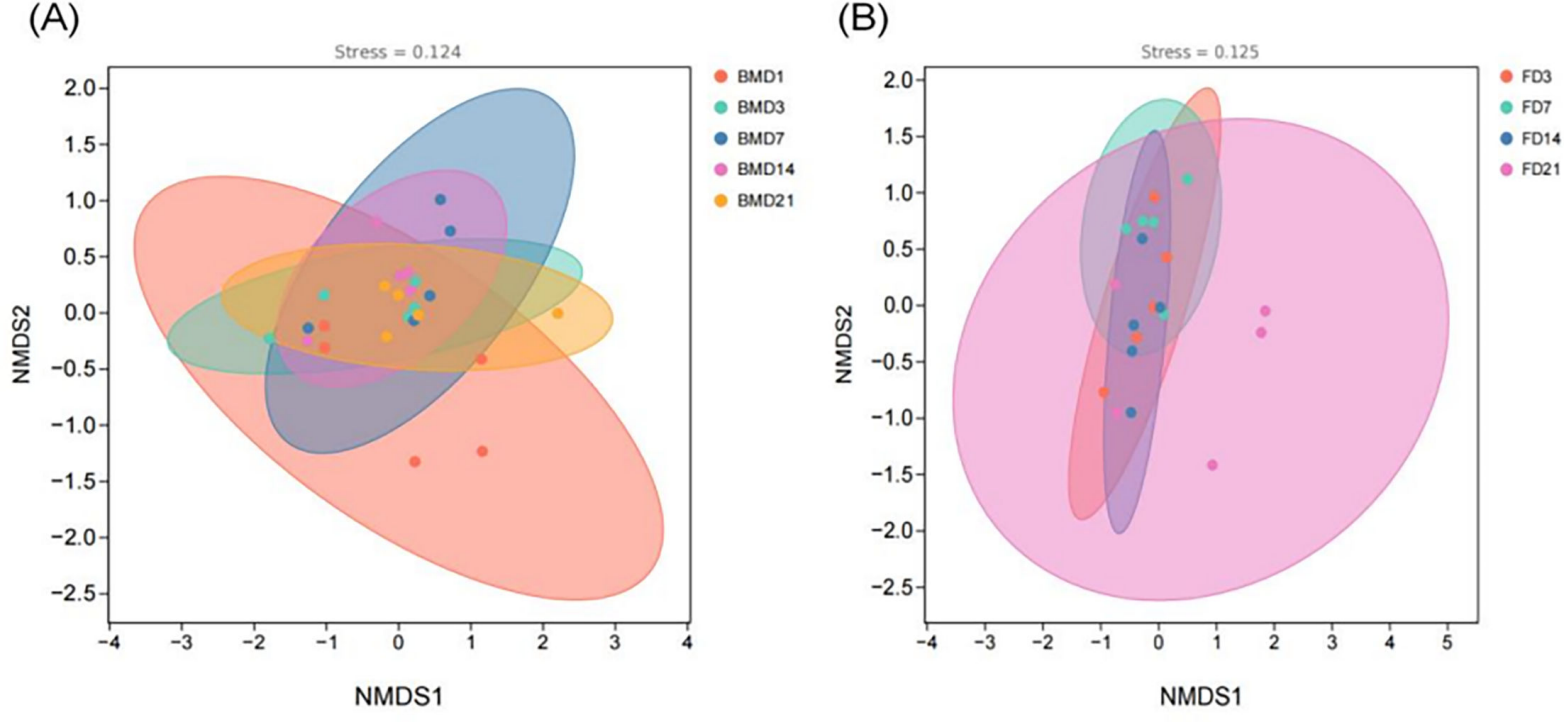
(**A**) β-diversity of breast milk microbiota. (**B**) β-diversity in rectal feces of goat kids. Use the Weighted-unifrac distance algorithm. NMDS results of the stress value (Stress), the smaller the better, it is generally believed that when the value is less than 0.2, the results of NMDS analysis are more reliable. Five per group per time point (n = 5).

To more visually illustrate the differences between each group, the NMDS analysis was performed in the present study. The results show that breast milk samples were significantly dispersed between the BMD1 group and the other four groups, which indicates that the microbial composition of breast milk in the BMD1 group differed significantly from that between the other four groups (Fig. 4A). The microbial community composition of goat kid feces was highly similar among the FD3, FD7, and FD14 groups, indicating a high degree of consistency in their microbial profiles. However, the samples from FD21 are more spatially dispersed than those from the three groups, FD3, FD7, and FD14, suggesting that the microbial composition of goat kid feces at FD21 differed significantly from that of the other groups (Fig. 4B).

### Microbiota composition analysis of microorganisms in breast milk and goat kid feces

**Fig 5:**
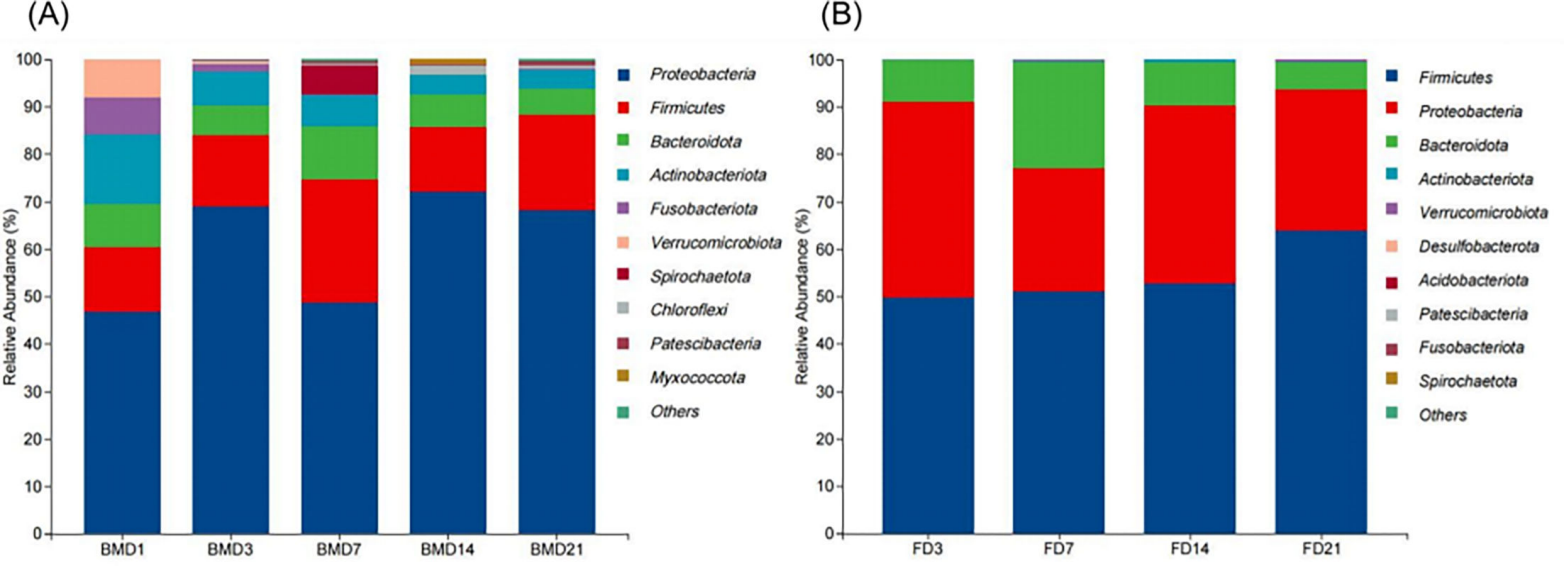
The composition of microbiota at the phylum level. (**A**) Composition of phylum-level microbiota in breast milk. (**B**) Composition of phylum-level microbiota in rectal feces of goat kids. Five per group per time point (n = 5).

The composition of breast milk microbiota at BMD1, BMD3, BMD7, BMD14, and BMD21 groups was dominated at the phylum level by *Proteobacteria* (46.71%, 69.00%, 48.84%, 72.00%, and 68.11%, respectively), *Firmicutes* (13.62%, 14.91%, 25.89%, 13.68%, and 20.18%, respectively), *Bacteroidota* (9.27%, 6.39%, 11.18%, 6.96%, and 5.43%, respectively), and *Actinobactenota* (14.59%, 7.19%, 6.53%, 4.06%, and 4.14%, respectively). The dominant bacterial phyla in goat kid feces at FD3, FD7, FD14, and FD21 groups were *Firmicutes* (49.85%, 51.15%, 52.77%, and 63.99%, respectively), *Proteobacteria* (41.34%, 25.96%, 37.42%, and 29.72%, respectively), and *Bacteroidota* (8.63%, 22.20%, 9.24%, and 5.69%, respectively). See attached Fig. 5.

**Fig 6:**
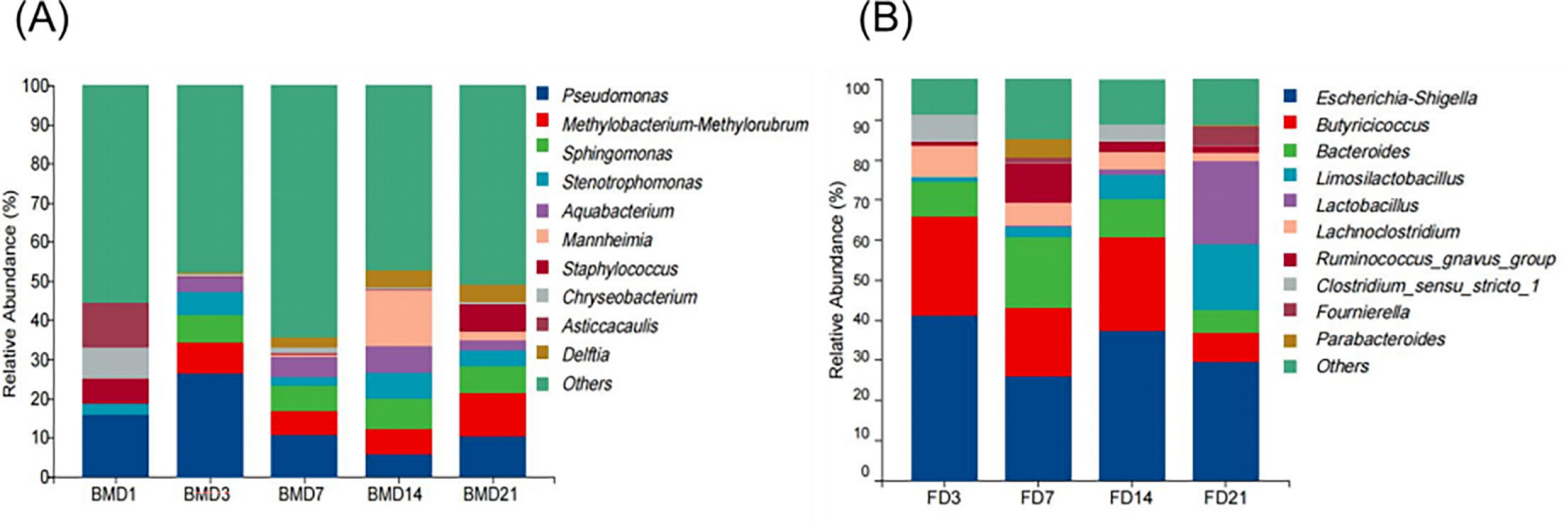
The composition of microbiota at the genus level. (**A**) Composition of genus-level microbiota in breast milk. (**B**) Composition of genus-level microbiome in rectal feces of goat kids. Five per group per time point (n = 5).

At the genus level, the most abundant bacteria in the BMD1 group of breast milk were *Pseudomonas*, *Asticcacaulis*, and *Chryseobacterium*. In later time points (BMD3, BMD7, BMD14, BMD21), genera, such as *Pseudomonas*, *Methylobacterium-Methylorubrum*, *Sphingomonas*, and *Staphylococcus,* became dominant (Fig.6A). In addition, we conducted statistical analyses using analysis of variance to assess differences in microbial genera across time points. Specifically, we found that *Pseudomonas* in breast milk showed significant differences (*P* < 0.05) between BMD3 and BMD14 (Table 3). The above results show that the predominant microbiota in breast milk varied at different ages. In addition, breast milk samples contained more soil and plant microbiota, in addition to respiratory, bacteremia, and meningitis-associated bacterial genera in this study. Similarly, the goat kid fecal microbiota shifted with age, with *Escherichia-Shigella*, *Butyricicoccus*, *Bacteroides*, *Lactobacillus*, and *Limosilactobacillus* being the predominant genera at various stages (Fig. 6B). There was a marginal trend toward differences in *Lactobacillus* abundance in rectal feces between the following time points: FD3 vs FD21 (*P* = 0.07), FD7 vs FD21 (*P* = 0.07), and FD14 vs FD21 (*P* = 0.10), although these differences did not reach statistical significance (*P* > 0.05) (Table 3). The presence of pathogenic and probiotic genera suggests a complex interplay between gut health and disease risk in early goat kid development.

**TABLE 3:** Significant results of microbial genera at different time points

Goat milk		
*Pseudomonas*	Significance	*P* value
BMD1 vs BMD3	ns^*b*^	0.58
BMD1 vs BMD7	ns	0.94
BMD1 vs BMD14	ns	0.59
BMD1 vs BMD21	ns	0.93
BMD3 vs BMD7	ns	0.17
BMD3 vs BMD14	*^a^	0.03
BMD3 vs BMD21	ns	0.16
BMD7 vs BMD14	ns	0.96
BMD7 vs BMD21	ns	>0.99
BMD14 vs BMD21	ns	0.96

Rectal feces of goat kids		
*Lactobacillus*	Significance	*P* value
FD3 vs FD7	ns	>0.99
FD3 vs FD14	ns	>0.99
FD3 vs FD21	ns	0.07
FD7 vs FD14	ns	>0.99
FD7 vs FD21	ns	0.07
FD14 vs FD21	ns	0.1

^*a*^ "*" indicates significant difference (*P <* 0.05).

^*b*^ ns, no significant difference (*P *> 0.05).

**Fig 7:**
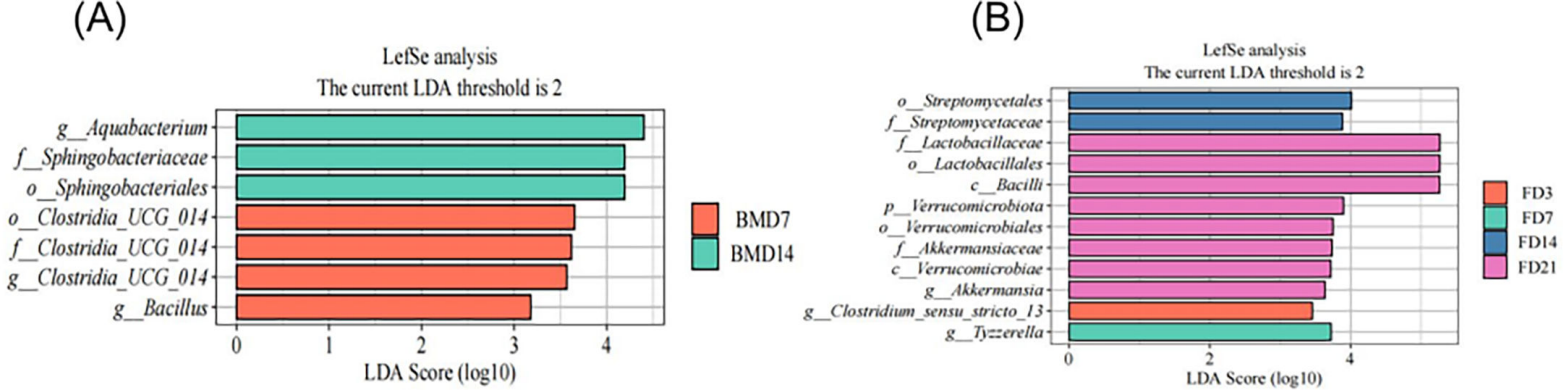
LEfSe analyses of microbiota. (**A**) LEfSe analysis of breast milk microbiota. (**B**) LEfSe analysis of rectal fecal microbiota in goat kids. LEfSe differed significantly at the genus level in distinguishing between breast milk microorganisms of different ages and rectal fecal microorganisms of goat kids of different ages. The genera in this figure were statistically significant (*P* < 0.05), and an LDA score ≥2.0 was considered a significant effect size. Five per group per time point (n = 5).

The analysis of differential bacteria in breast milk and goat kid feces samples across different ages using the LEfSe software. The results indicate that *Clostridia-ucg-014* and *Bacillus* were enriched in breast milk of the BMD7 group, while *Aquabacterium* and *Sphingobacteriaceae* were enriched in the BMD14 group. No significantly different species were identified in the other groups (Fig. 7A). The goat kid feces were enriched with *Clostridium sensu stricto-13* at the FD3 group, *Tyzzerella* was enriched at the FD7 group, *Streptomycetales* were enriched at the FD14 group, and *Lactobacillales*, *Bacilla*, *Verrucomicrobiota*, and *Akkermansia* were enriched at the FD21 group (Fig. 7B). This study found that as the lactation period in female goats extends, the microbiota in breast milk samples and in the intestines of goat kids becomes increasingly complex. Microbial diversity increased gradually, and the microbial community structure became more complex than that of the 0–7 days.

### Correlation analysis of breast milk microbiome with goat kid fecal microbiome

**Fig 8:**
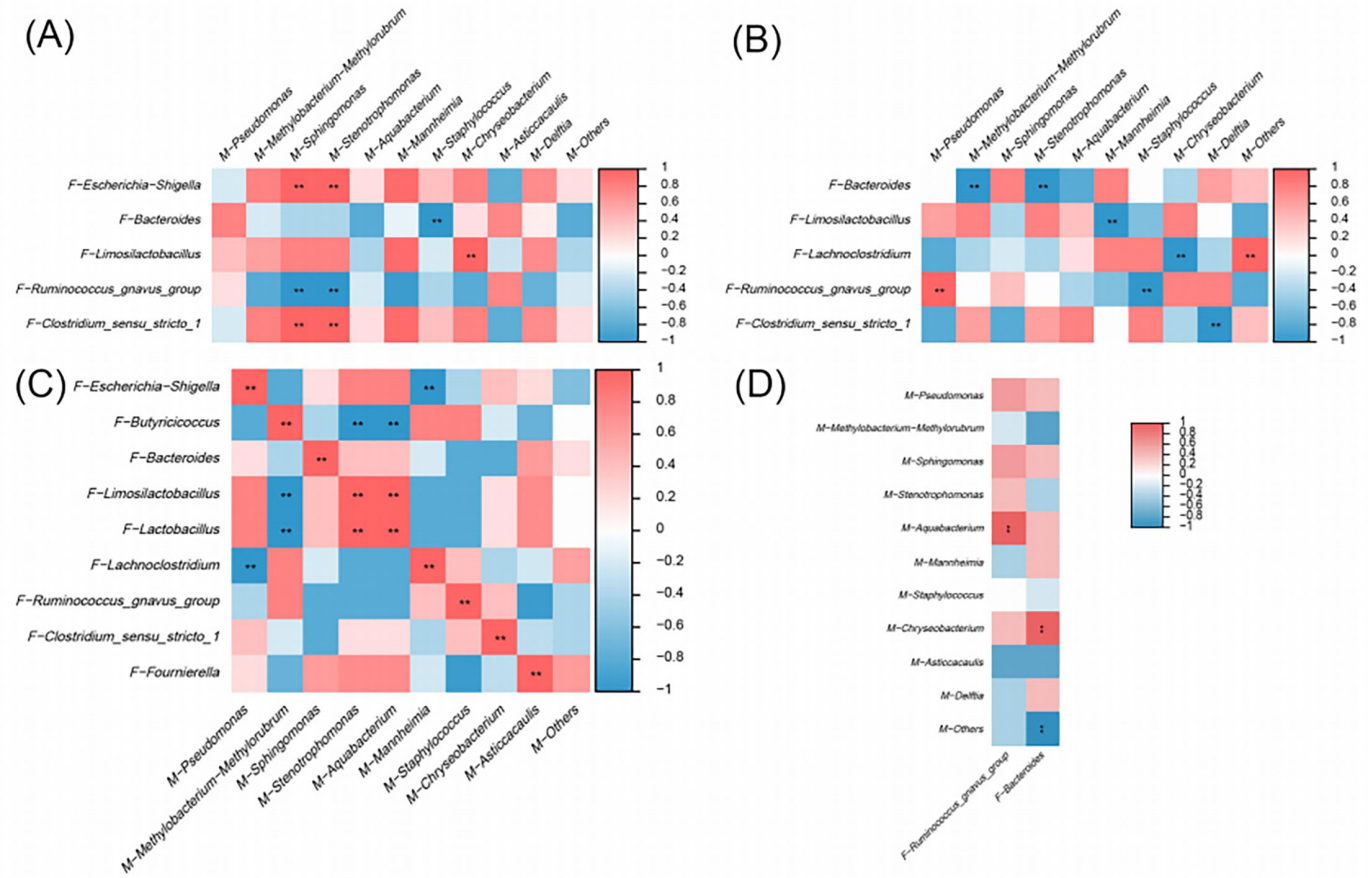
Heat map showing the correlation between breast milk microbiota and rectal fecal microbiota of goat kids. (**A**) Heatmap of the correlation between BMD3 and FD3 groups. (**B**) Heatmap of the correlation between BMD7 and FD7 groups. (**C**) Heatmap of the correlation between BMD14 and FD14 groups. (**D**) Heatmap of the correlation between BMD21 and FD21 groups. Color represents the correlation coefficient, with red representing a positive correlation and blue denoting a negative correlation. *P < 0.05, **P < 0.01, and ***P < 0.001.

**Fig 9:**
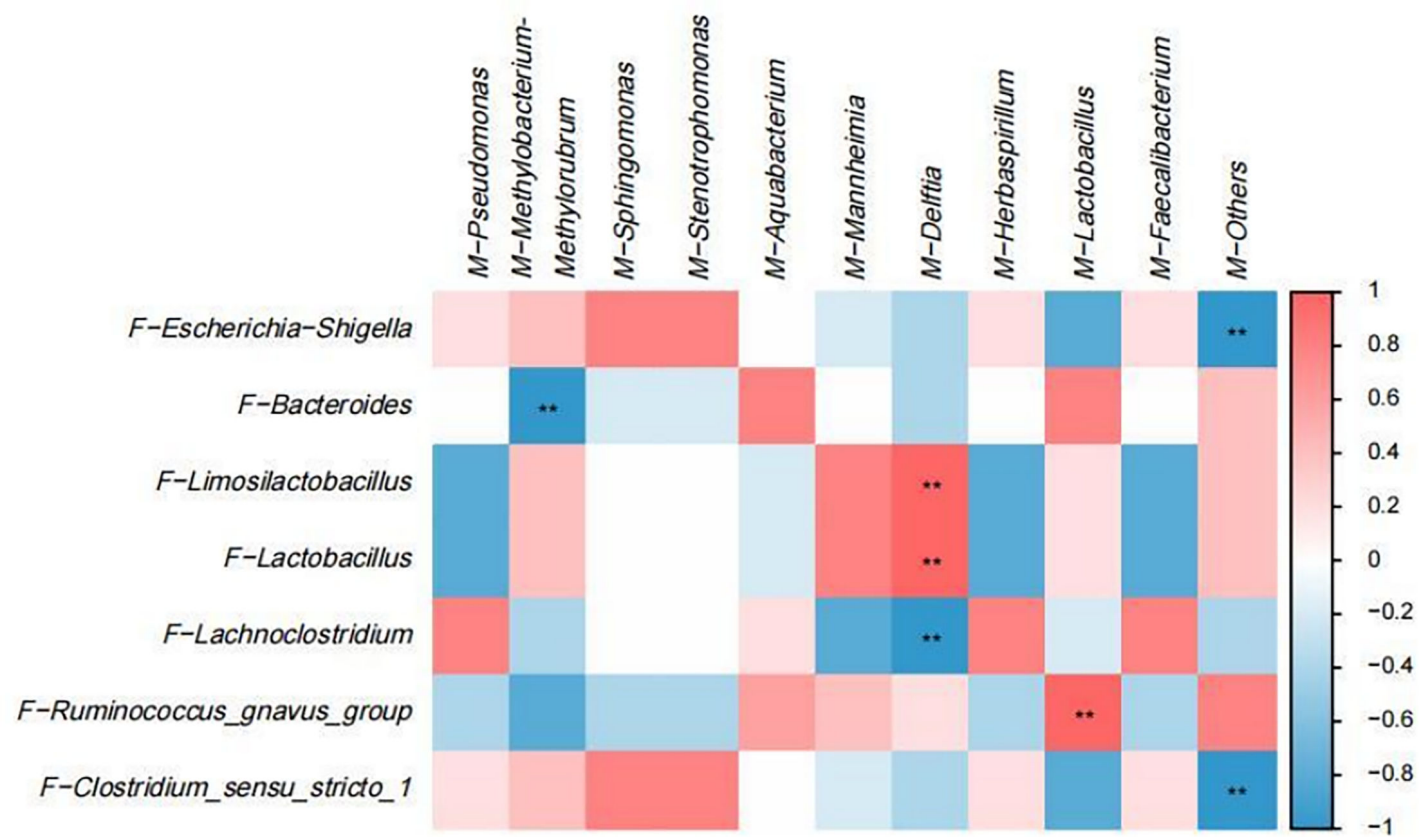
Heat map showing the correlation between breast milk microorganisms and rectal fecal microorganisms of goat kids. Color represents the correlation coefficient, with red representing a positive correlation and blue denoting a negative correlation. *P < 0.05, **P < 0.01, and ***P < 0.001.

We performed correlation analysis to explore the relationship between the breast milk microbiota and goat kid fecal microbiota (Fig. 8). Day 1 data were excluded from the correlation analysis because colostrum may not yet have affected the gut microbiota of goat kids. The analysis showed a positive correlation between *Escherichia-Shigella* and *Clostridium-sensu-stricto-1* in the rectal feces of goat kids in the FD3 group and *Sphingomonas* and *Stenotrophomonas* in the breast milk of the BMD3 group (Fig. 8A). *Bacteroides* in rectal feces of goat kids in the FD7 group were negatively correlated with *Methylobacterium-Methylorubrum* and *Stenotrophomonas* in breast milk of the BMD7 group (Fig. 8B). The correlation between *Limosilactobacillus* and *Mannheimia* is negative, as is the association between *Lachnoclostridium* and *Chryseobacterium. Ruminococcus_gnavus_group* was positively correlated with *Pseudomonas* and negatively correlated with *Staphylococcus* (Fig. 8B). In the FD14 group, the presence of *Escherichia-Shigella* in the rectal feces of goat kids was positively correlated with *Pseudomonas* in breast milk and negatively correlated with *Mannheimia*. *Butyricimonas* exhibited a positive correlation with *Methylobacterium-Methylorubrum*, while showing a negative correlation with both *Stenotrophomonas* and *Aquabacterium*. There is a positive correlation between *Ruminococcus_gnavus_group* and *Staphylococcus*, as well as between *Clostridium-sensu-stricto-1* and *Chryseobacterium*, and between *Fournierella* and *Asticcacaulis* (Fig. 8C). In the FD21 group, *Bacteroides* and *Ruminococcus_gnavus_group* in rectal feces of goat kids were positively correlated with *Chryseobacterium* and *Aquabacterium* in breast milk of the BMD21 group, respectively (Fig. 8D). In addition, the analysis showed that *Methylobacterium-Methylorubrum* and *Bacteroides* were negatively correlated, *Delftia* was positively correlated with *Limosilactobacillus* and *Lactobacillus*. In contrast, *Delftia* and *Lachnoclostridium* were negatively correlated. *Lactobacillus* was positively correlated with *Ruminococcus_gnavus_group* in breast milk. See attached Fig. 9.

### Probiotics in breast milk and rectal feces of goat kids

**Fig 10:**
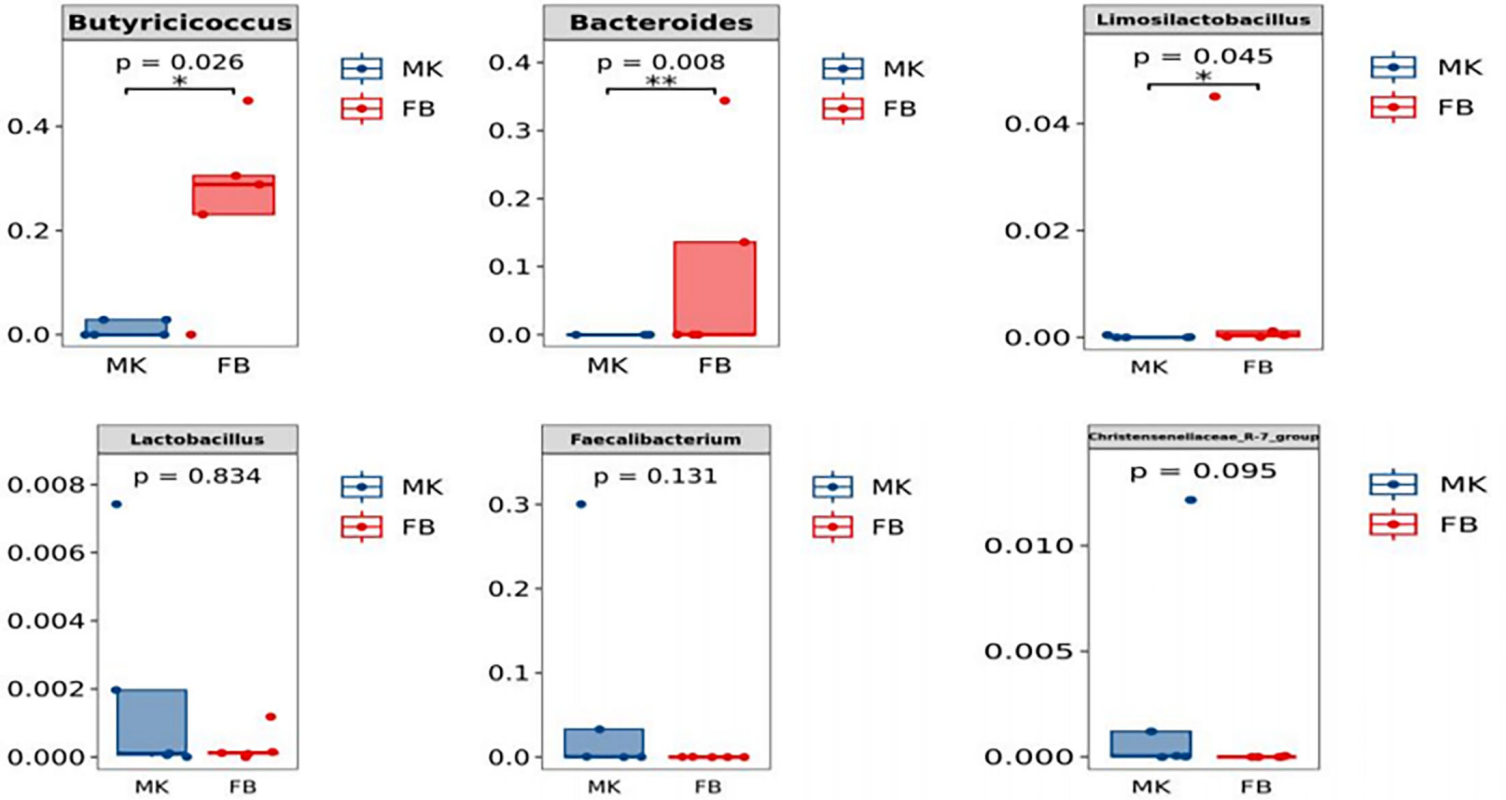
Probiotics and their abundance found in the rectal feces of goat kids and breast milk. MK: probiotics in breast milk; FB: probiotics in the rectal feces of goat kids. *P < 0.05, **P < 0.01, and ***P < 0.001.

Probiotic species and their abundance found in the breast milk and goat kid feces, as shown in Fig. 10. At the genus level, some of the known probiotics were found in breast milk and goat kid feces, including *Butyricicoccus*, *Bacteroides*, *Lactobacillus*, *Limosilactobacillus*, *Ruminococcus-gnavus-group*, and *Faecalibacterium*. The abundance of *Butyricoccus* (*P* < 0.05) and *Bacteroides* (*P* < 0.01) was significantly higher in goat kid feces than in breast milk, the abundance of *Limosilactobacillus* in goat kid feces was significantly higher in goat kid feces than in breast milk (*P* < 0.05), while the abundance of other probiotic genera in breast milk and goat kid feces was not significantly different (*P* > 0.05).

## DISCUSSION

The present study analyzed the composition and correlation of microorganisms in goat breast milk and their goat kid feces at different time periods using 16S rRNA high-throughput sequencing (V3–V4 region) in Sichuan black goats (China). In this study, we found that the diversity and abundance of microorganisms in breast milk changed irregularly with age. However, the reasons for this change are not clear and need to be further investigated. There was a significant difference in microbial diversity between colostrum and mature milk, indicating that the stage of lactation has a significant effect on the microbial community and diversity in breast milk (20). Goat kid gut microbes are exposed to the external environment suddenly after birth, which tends to complicate and diversify the intestinal microbiota of goat kids (21, 22). Previous studies have shown that the diversity of gastrointestinal microbiota in calves prior to weaning follows a non-linear trend, initially decreasing and then increasing, which is similar to the results of the present study between 7 and 21 days (23). The dynamics of intestinal microbial composition in goat kids with age are likely linked to the development of the intestinal environment. Specifically, the loss of certain microbiota may lead to the death of symbiotic bacteria.

At the phylum level, we found that the dominant phyla in breast milk samples of different days of age were *Proteobacteria*, *Firmicutes*, *Bacteroidetes*, and *Actinobacteria*, which is similar to the results of a previous study (24). Furthermore, Van et al. (25) found that *Firmicutes*, *Bacteroidetes,* and *Actinobacteria* were the dominant phyla in Holstein Friesian and Belgian Blue milk. *Proteobacteria*, *Firmicutes*, *Bacteroidetes*, and *Actinobacteria* are also the main gates of camel’s milk, but the specific role of bacteria in the microbial community of breast milk has not been clarified until now (26, 27). Our findings suggest that breast milk contains a diverse bacterial community, serving as a natural source of various microorganisms. Notably, the microbiota composition in BMD1 differed from other groups. In addition to the usual phyla (*Proteobacteria*, *Firmicutes*, *Bacteroidetes*, and *Actinobacteria*), *Verrucomicrobiota* and *Fusobacteriota* were found in higher abundance in colostrum (BMD1 and BMD3). This suggests that colostrum microbiota provide essential nutrients, such as proteins, lipids, vitamins, lactose, minerals, hormones, enzymes, peptides, antimicrobial substances, anti-inflammatory mediators, and growth factors. These components support the growth and gastrointestinal development of neonatal goat kids and play a key role in the early maturation of the goat kid’s gastrointestinal tract (14, 24). Our results also show that different stages of lactation influence the predominant phyla in breast milk. Notably, the higher abundance of *Actinobacteria* in goat milk may influence early gut microbial colonization in goat kids through vertical transmission, potentially increasing the risk of disease (28, 29).

*Firmicutes*, *Proteobacteria*, and *Bacteroidetes* predominated in the goat kid feces of different ages. Interestingly, the abundance of *Firmicutes* increased after 14 days of age, whereas the opposite was true for *Proteobacteria* and *Bacteroidetes*, possibly related to the ingestion of solids by the goat kids. A previous study has shown that the *Firmicutes* contains mainly butyrate-producing bacteria, which act to maintain the integrity of the intestinal barrier and are involved in the digestion, absorption, and metabolic transport of nutrients (30, 31). Therefore, a higher abundance of *Firmicutes* can help goat kids to digest plant-based feeds and improve their adaptation to roughage.

Although goat’s milk is rich in nutrients, it is easily contaminated by microorganisms that may cause disease in goat kids (32). In this study, we observed the predominance of *Pseudomonas* and *Staphylococcus* in breast milk samples. Scatamburlo et al. (32) reported that *Pseudomonas* spp. are ubiquitous in dairy farms (32). Additionally, studies on *Staphylococcus* spp. in milk from healthy animals have shown that non-*Staphylococcus aureus* strains and mammalian cocci are among the most frequently isolated bacteria (33). These findings suggest that *Pseudomonas* and *Staphylococcus* are prevalent in the milk of healthy animals. However, further research is needed to understand their impact on goat kid health and their potential pathogenicity. *Pseudomonas aeruginosa*, in particular, is associated with milk spoilage and gastrointestinal disturbances (34, 35). The presence of *Staphylococcus* also raises concerns, as it is a known cause of mastitis and foodborne illnesses (36–38). These findings underscore the importance of monitoring and controlling bacterial contamination in goat milk production to improve animal health and product quality. Moreover, bacteria of the genus *Mannheimia* are recognized as pathogens that can cause severe respiratory disease and mastitis in goats. However, in the present study, all goats were confirmed to be healthy, and SCC measurements were consistently within the normal range, effectively ruling out subclinical mastitis. The transient presence of *Mannheimia* in the BMD7 milk samples may represent a non-pathogenic colonization event, as members of this genus can be part of the commensal microbiota in healthy animals. This observation underscores the microbial diversity present in breast milk even under healthy physiological conditions and suggests that not all occurrences of potentially pathogenic genera indicate disease.

*Escherichia-Shigella* is widely distributed in the intestinal tracts of goats. Although they are often considered potential pathogens, some members of the genus *E. coli* can help anaerobic bacteria create an anaerobic environment by consuming oxygen, thus establishing a suitable living environment for microaerobic and anaerobic bacteria, such as *Lactobacillus*, to colonize. Studies have shown that *Lactobacillus* is a major member of the initially colonizing bacteria in the intestines of healthy newborn offspring (39), which corresponds with the results of the present study. The *Butynicicoccus* genus is predominantly isolated from animal and human fecal matter. Its primary metabolite, butyric acid, is absorbed by intestinal epithelial cells and serves as an essential energy substrate through fatty acid oxidation. Importantly, butyric acid is intrinsically linked to overall health, with significant implications for intestinal homeostasis, inflammation modulation, immune function enhancement, and antioxidant activity (40, 41). The majority of *Bacteroides* species are prolific producers of acetate and propionate, exerting significant metabolic roles within the intestinal environment (42). *Limosilactobacillus* and *Lactobacillus*, recognized as beneficial commensals within the intestinal tract, serve a pivotal nutritive function. These bacteria effectively modulate the intestinal microecological equilibrium, sustain the normal physiological functions of the gastrointestinal tract, and contribute to the enhancement of systemic immunity in the host. Additionally, they fortify the host’s defenses against the incursion of exogenous pathogenic microorganisms by facilitating the secretion of cytokines (43, 44). Interestingly, a trend of decreasing abundance with age was observed for *Lachnoclostridium*, while some probiotic bacteria (including *Limosilactobacillus* and *Lactobacillus*) exhibited an increasing trend. These results strongly suggest that probiotics play a significant role in the establishment of the early intestinal microbiota in goat kids.

Gut microbial colonization early in life is critical for offspring growth and development. In humans, it has been established that the gut microbiota of infants is primarily inherited from their mothers, with vertical transmission occurring through the gut, the birth canal, and breast milk (45, 46). Generally, goat kids are predominantly reliant on breast milk during the initial month of life, which serves as the principal source of nutrition. Thus, the current study further analyzed the correlation between breast milk and the rectal fecal microbiota of goat kids and found that the microorganisms in breast milk and the microorganisms in the rectal feces of goat kids had a reciprocal relationship. There was a positive correlation between *Sphingomonas*, *Stenotrophomonas*, and *Pseudomonas* in breast milk and *Escherichia-Shigella* in the rectal feces of goat kids. Studies have shown that *Sphingomonas*, *Stenotrophomonas,* and *Clostridium-sensu-stricto-1* are conditional pathogens (30, 38). Especially, *Pseudomonas* and *Escherichia-Shigella* can cause diarrhea (27, 32). In addition, *Sphingomonas*, *Stenotrophomonas*, and *Pseudomonas* in breast milk were positively correlated with probiotic genera in the rectal feces of goat kids. This suggests that certain species may favor the growth of probiotics. There are complex interactions between conditionally pathogenic bacteria in breast milk, such as *Staphylococcus* and *Pseudomonas*, and probiotics, and under normal circumstances, these interactions are beneficial. These include competition, synergy, symbiosis, and parasitism, which promote the growth of probiotics and contribute to the maintenance of microbial diversity (47). In summary, the study shows that microorganisms present in breast milk significantly influence the microbial colonization of the intestinal tract in goat kids through vertical transmission. Notably, such as *Lactobacillus*, exert a pivotal role in fostering early intestinal health in animals (17).

The traditional perspective posits that breast milk is sterile; however, recent studies have stood in the opposite direction and believe that breast milk is rich in a wide range of microorganisms. These microorganisms provide a constant supply of commensal, mutualistic, and potentially probiotics to infants (15). Research indicates that the microbiota in breast milk plays an important role in the colonization of infant gut microbes and the development of the neonatal immune system (48). *Bacteroides*, *Limosilactobacillus*, and *Lactobacillus*, recognized as probiotics, constitute crucial genera in the manufacturing of dairy products. Notably, certain bacterial strains within these genera have been implicated in immunomodulatory processes within the host organism (49). *Lactobacillus* and *Butyricicoccus* are hypothesized to exhibit heightened anti-infective and reparative properties, potentially contributing to the amelioration of postpartum mastitis in goats (50). In this study, we identified common probiotics, including *Limosilactobacillus* and *Lactobacillus*, in both goat breast milk and goat kid feces. This discovery strongly suggests that the bacteria present in breast milk can be detectable in the intestinal microbiota of goat kids and a potential transmission of bacteria from goat milk to its offspring’s gut and further colonization. Due to the limited resolution of 16S rRNA sequencing technology, it is essential to undertake additional research to identify specific probiotic strains within breast milk and assess their functional roles in promoting goat kid health and growth. Moreover, exploring the mechanisms behind vertical microbial transmission and the environmental factors influencing the breast milk microbiota could provide valuable insights for improving goat kid rearing practices.

### Conclusion

This study employed 16S rRNA gene sequencing and identified significant variability in the microbial community structure of breast milk and goat kid rectal feces at different ages. Correlation analysis suggested that microbes from breast milk colonize the goat kid’s gastrointestinal tract through vertical transmission, influencing the development of the early gut microbiota in goat kids. Notably, key probiotic genera in breast milk, such as *Lactobacillus*, *Limosilactobacillus*, and *Ruminococcus-gnavus-group,* have a substantial impact on the intestinal microbiota of goat kids. Additionally, further research into the mechanisms of probiotic action can provide information on available strains for early intervention in goat kids.
